# Individual placement and support: cross-sectional study of equality of access and outcome for Black, Asian and minority ethnic communities

**DOI:** 10.1192/bjb.2021.9

**Published:** 2022-02

**Authors:** Rachel Perkins, Rash Patel, Amelia Willett, Laura Chisholm, Miles Rinaldi

**Affiliations:** 1 Implementing Recovery through Organisational Change (ImROC), Nottingham, UK; 2 Central & North West London NHS Foundation Trust, London, UK; 3 Working Well Trust, London, UK; 4 South West London and St George's Mental Health NHS Trust, London, UK; 5 Nordland Hospital Trust, Centre for Work and Mental Health, Bodø, Norway

**Keywords:** Supported employment, ethnicity, serious mental illness, individual placement and support, Black, Asian and minority ethnic communities

## Abstract

**Aims and method:**

To explore whether people from Black, Asian and minority ethnic (BAME) communities experience equality of access and outcome in individual placement and support (IPS) employment services. Cross-sectional data were analysed of all people with severe mental health problems who accessed two mature high-fidelity IPS services in London in 2019 (*n* = 779 people).

**Results:**

There were no significant differences between the proportions of people who gained employment. The data strongly suggest that people from BAME communities are not differentially disadvantaged in relation to either access to or outcomes of IPS employment services.

**Clinical implications:**

The challenge for mental health professionals is not to decide who can and who cannot work but, how to support people on their case-loads to access IPS and move forward with life beyond their illness.

It is recognised that people from Black, Asian and minority ethnic (BAME) communities both perceive^1^ and experience inequality in access,^2^ experience and outcomes^3^ within mental health services in England. The NHS Long Term Plan makes a renewed commitment to improve and widen access for adults needing mental health support partly through a new community-based offer. This includes access to employment support for people with severe mental health problems through the expansion of the evidence-based individual placement and support (IPS) approach.^4^

IPS involves a direct, individualised search for competitive employment that avoids lengthy pre-employment preparation or training and does not screen people for work ‘readiness’. It does not exclude people on the basis of diagnosis, symptoms or substance misuse. IPS is a ‘place and train’ approach, rather than a traditional ‘train and place’ approach, to vocational rehabilitation. There are now 27 randomised controlled trials supporting the efficacy of IPS compared with traditional vocational rehabilitation.^5^ National Institute for Health and Care Excellence (NICE) guidance recommends IPS for people with severe mental health problems who wish to gain and retain employment.^6,7^

Within this context, consideration needs to be given to ensuring that people from BAME communities have fair access to employment support and equal opportunities to gain employment. There has been considerable concern that there is no parity between BAME communities and the White majority in access, experience and outcomes of mental healthcare^8^ and so it is right that we ask questions about the effectiveness of IPS for BAME groups.

## Method

This was a service evaluation of two IPS services in London. Formal ethical approval was therefore not required.

### The IPS services

To explore whether people from BAME communities experience equality of access and outcome in IPS, two IPS services together serving five London boroughs were selected:

- Central and North West London NHS Foundation Trust (CNWL) IPS service, serving people using secondary adult community mental health services in the boroughs of Westminster, Kensington & Chelsea, Harrow and Hillingdon (CNWL also provides IPS services in Milton Keynes but data from this service were not included in the current study because this was a relatively new service and our focus was on mature London services);
- Working Well Trust (WWT) IPS service, which works in partnership with East London NHS Foundation Trust (ELFT) serving people using secondary adult community mental health services in the borough of Tower Hamlets.

These services were selected because they are both mature, high-fidelity IPS services recognised as centres of excellence by the Centre for Mental Health.^9^ Both serve areas of London where there are large BAME communities and they represent the two main models of providing IPS in English secondary mental healthcare services. The CNWL IPS service is provided by the trust and all employment specialists are trust employees and members of multidisciplinary adult community mental health teams. The WWT IPS service is a voluntary sector organisation whose employment specialists are employed by the WWT but integrated into ELFT Tower Hamlets adult community mental health teams.

### Data

The two IPS services provided anonymised cross-sectional data on all people with severe mental health problems who accessed their service between 1 January and 31 December 2019. For each person, the following anonymised data were supplied:

- ethnicity (White, Asian/Asian British, Black/Black British, Mixed, Other)
- age
- gender
- date of accessing the IPS service
- job outcome (whether the person gained at least one day of open paid employment by 31 December 2019). More detailed data on duration of employment were not available but anecdotal evidence suggests that in practice the majority were employed for a longer period.

Data were also provided by the respective mental health trusts on the ethnicity and gender breakdown of everyone served by their adult community mental health teams for the same time period.

## Results

Table 1 shows that *n* = 779 people accessed the two IPS services between 1 January 2019 and 31 December 2019: *n* = 412 accessed the CNWL IPS service and *n* = 367 accessed the WWT IPS service. Ethnicity data were available for *n* = 714 (92%) of these people.

**Table 1:** Number of people accessing the individual placement and support (IPS) services between 1 January and 31 December 2019, by ethnicity, gender and age

	CNWL IPS service	WWT IPS service	Total
People accessing IPS 1 Jan to 31 Dec 2019, *n*	412	367	779
Ethnicity, *n* (%)			
White	134 (36.7)	114 (41.3)	278 (38.9)
Asian/Asian British	77 (21.1)	114 (32.7)	191 (26.8)
Black/Black British	64 (17.5)	63 (18.1)	127 (17.8)
Mixed	38 (10.4)	21 (6.0)	59 (8.3)
Other	52 (14.2)	7 (2.0)	59 (8.3)
Missing (not known/prefer not to say), *n*	47	18	65
Gender, *n* (%)			
Male	207 (50.2)	180 (49.2)	387 (49.7)
Female	205 (49.8)	186 (50.8)	391 (50.3)
Missing (not known/prefer not to say), *n*	0	1	1
Age, years: mean (s.d.), range	37.73 (12.02), 17–65	34.39 (10.74), 18–69	36.16 (11.55), 17–69

CNWL, Central and North West London NHS Foundation Trust; WWT, Working Well Trust.

### Equality of access

To explore equality of access to IPS services for those from BAME communities, for each service the number of people of different ethnic communities accessing the service was compared with the ethnic breakdown of those using secondary adult community mental health services in the boroughs served, using a *χ*^2^-test statistic for goodness of fit. The ethnic breakdown of those using secondary adult community mental health services was selected rather than general population data for the boroughs served because it is people using secondary adult community mental health services who constitute the population eligible to access the IPS service. The results of these analyses can be seen in Table 2.

**Table 2:** Proportion of people from different BAME communities accessing individual placement and support (IPS) services in 2019 in comparison with their proportions in the population using adult community mental health services

	People accessing IPS service, *n* (%)	People using adult community mental health services, *n* (%)	People from BAME communities accessing IPS services, %	
CNWL IPS service				
White	134 (36.7)	2774 (39.6)	4.8	*χ*^2^ = 22.05, *P* < 0.01
Asian/Asian British	77 (21.1)	1158 (16.5)	6.6	
Black/Black British	64 (17.5)	810 (11.5)	7.9	
Mixed	38 (10.4)	936 (13.3)	4.1	
Other	52 (14.2)	1335 (19.0)	3.9	
Not known/stated	47 (11.4)	1350 (16.1)	3.5	
WWT IPS service				
White	144 (41.3)	900 (41.1)	1.6	*χ*^2^ = 34.22, *P* < 0.01
Asian/Asian British	114 (32.7)	805 (36.7)	14.2	
Black/Black British	63 (18.0)	259 (11.8)	24.3	
Mixed	21 (6.0)	61 (2.3)	34.4	
Other	7 (2.0)	167 (7.6)	4.2	
Not known/stated	18 (4.9)	90 (3.9)	20.0	

BAME, Black, Asian and minority ethnic; CNWL, Central and North West London NHS Foundation Trust; WWT, Working Well Trust.

Table 2 shows that there were significant differences in the proportions of people of different ethnicities accessing the IPS service compared with the respective secondary adult mental health service populations (CNWL IPS service: *χ*^2^ = 22.05, *P* < 0.01; WW IPS Service *χ*^2^ = 34.22, *P* < 0.01).

In both services, the proportions of White and Asian/Asian British clients accessing IPS were similar to those in the population of people using secondary adult community mental health community services. However, in both services, the proportion of Black/Black British clients accessing IPS was 52% greater than in the population using secondary adult community mental health services: respectively 17.5% compared with 11.5% in the CNWL IPS service and 18.0% compared with 11.8% in the WWT service.

The proportions of men and women accessing the IPS service did not differ significantly from the proportions using adult community mental health services (CNWL: *χ*^2^ = 2.61, *P* = 0.11; Working Well Trust: *χ*^2^ = 0.14, *P* = 0.71).

### Equality of outcome

To explore equality of employment outcome of IPS services for those from BAME communities, job outcomes for people of different ethnicities were compared using *χ*^2^-test statistics. Two separate analyses were performed. The first considered everyone accessing the IPS services between 1 January and 31 December 2019 and whether or not they had gained employment by 31 December 2019. However, some of these people – those accessing the service later in the year – would only have had the opportunity for a very short period of support before 31 December 2019. Therefore, a separate analysis was conducted considering only those who had accessed the service in the first half of the year (between 1 January and 30 June 2019) and had therefore had the opportunity of at least 6 months’ support. The results of these analyses can be seen in Table 3.

**Table 3:** Job outcomes by 31 December 2019 by ethnic group

	People who gained employment by 31 Dec 2019, *n* (%)		
	CNWL IPS service	WWT IPS service	Total
All who accessed IPS 1 Jan to 31 Dec 2019	141 (38.6) (412 people, ethnicity data available for 365)	107 (30.7) (367 people, ethnicity data available for 349)	248 (34.7) (779 people, ethnicity data available for 714)
Ethnicity			
White	47 (35.1)	46 (31.9)	93 (33.5)
Asian/Asian British	35 (45.5)	37 (32.5)	72 (37.7)
Black/Black British	23 (35.9)	18 (28.6)	41 (32.3)
Mixed	15 (39.5)	5 (23.8)	20 (33.9)
Other	21 (40.4)	1 (14.3)	22 (37.3)
	*χ*^2^ = 2.50, *P* = 0.64	*χ*^2^ = 1.76, *P* = 0.78	*χ*^2^ = 1.46, *P* = 0.84
People who had opportunity for at least 6 months’ support by 31 Dec 2019^a^	55 (43.3) (139 people, ethnicity data available for 127)	54 (42.2) (135 people, ethnicity data available for 128)	109 (42.7) (274 people, ethnicity data available for 255)
Ethnicity			
White	18 (39.1)	29 (46.0)	47 (43.1)
Asian/Asian British	8 (40.0)	13 (39.4)	21 (39.6)
Black/Black British	6 (30.0)	10 (45.5)	16 (38.1)
Mixed	10 (66.7)	2 (22.2)	12 (50.0)
Other	13 (50.0)	0 (0)	13 (48.1)
	*χ*^2^ = 5.66, *P* = 0.22	*χ*^2^ = 2.78, *P* = 0.59	*χ*^2^ = 1.43, *P* = 0.84

CNWL, Central and North West London NHS Foundation Trust; IPS, individual placement and support; WWT, Working Well Trust.

a. i.e. accessed IPS between 1 January and 30 June 2019.

Table 3 shows that, when considering everyone accessing the service between 1 January and 31 December 2019, 34.7% had gained employment by 31 December 2019: 38.6% in the CNWL IPS service and 30.7%% in the WW IPS Service. Considering only those who had the opportunity of at least 6 months’ IPS support (those who had accessed the service between 1 January and 30 June 2019), by 31 December, 42.7% had gained employment: 43.3% in the CNWL IPS service and 42.2% in the WW IPS service.

There was no significant difference in the employment outcomes by 31 December 2019 for people from different ethnic backgrounds (for everyone accessing IPS between 1 January and 31 December 2019: *χ*^2^ = 1.46, *P* = 0.84; for those who had the opportunity for at least 6 months’ input: *χ*^2^ = 1.43, *P* = 0.84). Neither was there any significant difference between the outcomes for people of different ethnicities in either of the services when considered separately.

This equality of employment outcome was found when men and women from different ethnic backgrounds were considered separately. For those accessing IPS between 1 January and 31 December 2019 there was no significant difference in job outcomes between those from different ethnic groups for men (*χ*^2^ = 7.62, *P* = 0.11) or for women (*χ*^2^ = 2.84, *P* = 0.59). Similarly, equality of employment outcome was found for people from different ethnic backgrounds in different age groups (up to 25 years of age: *χ*^2^ = 1.62, *P* = 0.81; 26–40 years: *χ*^2^ = 4.38, *P* = 0.38; 41–55 years: *χ*^2^ = 0.50, *P* = 0.97; over 55 years: *χ*^2^ = 2.93, *P* = 0.60).

## Discussion

The data collected from these two mature high-fidelity London IPS services strongly suggest that IPS is equally effective in securing employment for people of different ethnic backgrounds using secondary mental health services. For men and women, young and old there were no significant differences between the proportions who gained employment by the end of the year in which they accessed IPS services. It is not known whether similar results would be obtained in less well-established services – it takes time to implement an effective IPS service. With the NHS England national roll-out of IPS it is essential that routine service monitoring includes access and outcome data broken down by ethnicity to demonstrate the key IPS principle of ‘zero exclusion’ that ensures services are equally effective across different communities.

Although the proportions of men and women accessing these IPS services did not differ, there were significant differences in the proportions of people of different ethnicities accessing them. These differences do not suggest differential disadvantage for people from BAME communities. Quite the reverse, the proportions of Black/Black British people accessing each IPS services were higher than their proportions in the populations of people using secondary adult community mental health services in the areas (CNWL: 17.5 *v.* 11.5%; WWT: 18.0 *v.* 11.8%). However, there are marked differences between the two services: in CNWL the proportion of people from different ethnic communities accessing IPS did not differ markedly, but at WWT there were substantial differences. The reasons for this cannot be ascertained from the data. For example, it may reflect a positive bias in referrals to IPS or a greater interest in work opportunities by the different ethnic communities (perhaps itself reflecting greater deprivation/different employment rates). The data considered here are for those who engaged with the services: it is not known how many were referred but did not engage with the service offered. It should also be noted that the ‘not known/stated’ ethnicity category was higher in CNWL than in WWT/Tower Hamlets adult mental health services.

It has sometimes been suggested that South Asian communities may be protective of people with psychosis and consider employment as a risk. Our study would suggest that this is not the case. It showed no differences in access or outcome for Asian/Asian British people. Similarly, previous research has demonstrated that Asian/Asian British people using IPS services were more likely to be in employment than their White counterparts.^10^ However, in our study it should also be noted that, although in CNWL the proportion of Asian/Asian British people was substantially higher among those accessing IPS services than among the adult community mental health services population (21.1 *v.* 16.5%), in WWT it was lower (32.7 *v.* 36.7%). It is possible that this difference results from different composition of the Asian/Asian British population (WWT: 80.5% Bangladeshi, 4.9% Indian, 4.1% Pakistani; CNWL: 4.8% Bangladeshi, 41.7% Indian, 13.6% Pakistani). Clearly this area requires greater understanding and a more detailed breakdown of ethnicity than was possible here.

Literature relating to BAME communities and mental health services is replete with examples of disparities in access, experience and outcome of services and, in particular, high levels of compulsion.^11^ In England, people with mental health problems from BAME communities have been less likely to use employment support services and as a consequence have been less likely to succeed in gaining employment than their White British peers.^12,13^ Morgan et al^14^ have suggested that addressing the social needs of BAME patients is likely to lead to improved clinical outcomes and engagement with services. Perhaps increasing the availability of IPS is one good way of doing this?

### How IPS works

IPS services are entirely voluntary. In line with the fidelity standards for IPS,^15^ an employment specialist is integrated into a clinical team. People using secondary mental health services can access IPS services if they themselves want to work – there is no selection on the basis of diagnosis or supposed ‘readiness’ for work. IPS is personalised and based on the individual's preferences and choices – very different from typical mainstream employment support programmes. Through shared decision-making, IPS rebalances power and encourages a collaborative dialogue between the employment specialist and the individual. Shared decision-making relies on two sources of expertise: the employment specialist as an expert on supporting individuals with mental health problems to gain and retain employment, and the individual as an expert on themselves, their social circumstances, attitudes to work, and health, values and preferences. Both must be willing to share information and accept responsibility for joint decision-making. The employment specialist needs to provide information about the most effective ways to gain and retain employment. The individual needs to tell the employment specialist about their preferences. As IPS is integrated into the clinical team, the challenge for mental health professionals is not to decide who can and who cannot work but how to support people on their case-loads to access IPS and move forward with life beyond their illness.^16^ Two interesting findings arise from this study: a disproportionate number of Black/Black British people were attracted to the IPS services – gaining employment was of importance to them – and there were no significant differences in outcomes for people from different ethnic backgrounds.

### Limitations and implications

Clearly, further research is necessary. The naturalistic design of this study is a limitation yet provides a real-world understanding of access to and outcomes from IPS services achieved for BAME communities using secondary mental health services. The data collected here considered only outcomes at the end of the year studied. It is possible that others would have gone on to gain employment had longer-term follow-up been possible. Data on type of employment and job tenure were not collected, neither could people's experience of using the services be ascertained, and a more detailed breakdown of ethnicity than was possible here would clearly be desirable. However, it is interesting to note that, of the three randomised controlled trials of IPS in England, none has reported outcomes by ethnicity,^17–19^ whereas some of the naturalistic studies have.^20,21^ Although there is a clear need for better quantitative data, the collection of qualitative data relating to people's experience of using IPS services is necessary to understand some of the differences found and ensure equality of access and outcome for all.

Everyone has the right to be treated with dignity and respect, without discrimination, and to be able to access appropriate mental healthcare when it is needed. Identifying and reducing health inequalities in access, experience and outcomes is essential to the delivery of high-quality mental healthcare. Mental health services have a duty to use data and existing resources to identify inequalities. The present study strongly suggests that people from BAME communities are not differentially disadvantaged in relation to either access to or outcomes of IPS employment support services.

## Data Availability

Data are available from the corresponding author.

## About the authors

**Rachel Perkins** is a clinical psychologist and senior consultant at Implementing Recovery through Organisational Change (ImROC), based in London, UK. **Rash Patel** is Head of Employment and Volunteering at Central and North West London NHS Foundation Trust, London, UK. **Amelia Willett** is Operations Director at the Working Well Trust, London, UK. **Laura Chisholm** is Employment Services Manager at the Working Well Trust, London, UK. **Miles Rinaldi** is Head of Strategic Development at South West London and St George's Mental Health NHS Trust, London, UK.
